# Comparison of endovascular procedures of the lower extremities and iliac arteries with and without navigation assistance Vascular Navigation PAD: study protocol for a prospective multicenter randomized clinical trial

**DOI:** 10.1186/s13063-026-09841-x

**Published:** 2026-06-15

**Authors:** Stephan Masius, Sebastian Zerwes, Alexander Gombert, Christian Uhl, Alexander Hyhlik-Dürr

**Affiliations:** 1 Gefäßchirurgie, Medizinische Fakultät, Universität Augsburg, Stenglinstr. 2, Augsburg, 86156 Germany; 2 Universitätsklinikum Aachen, RWTH Aachen, Pauwlsstraße 30, Aachen, 52074 Germany

**Keywords:** Peripheral artery disease, Navigation, C-arm

## Abstract

**Background:**

Endovascular procedures using a mobile C-arm are commonly performed in patients with peripheral artery disease. During these procedures, digital subtraction angiography (DSA) requires intra-arterial administration of contrast agent and exposes both patients and staff to radiation from the C-arm. The Vascular Navigation PAD system (Brainlab, Germany) may reduce the number of DSAs performed and thereby decrease both contrast agent use and radiation exposure. The objective of this study is to evaluate the safety and clinical performance of the Vascular Navigation PAD system compared with standard care in endovascular PAD treatment. The primary aim is to determine whether the device reduces the volume of contrast agent used during lower-extremity procedures. Secondary objectives include assessing patient and physician radiation exposure, patient air kerma, and the device’s ability to support accurate endovascular navigation.

**Methods:**

This is a multicenter, prospective, randomized confirmatory trial comparing navigation-assisted procedures with conventional procedures without navigation assistance. Patients allocated to the intervention group will undergo treatment using Vascular Navigation PAD in addition to a mobile C-arm. A total of 160 patients will be randomized in a 1:1 ratio, with 40 patients per group enrolled at each of the two participating hospitals. The primary endpoint is contrast agent volume. Secondary endpoints include patient radiation exposure, physician radiation exposure, fluoroscopy time, and the number of DSAs performed per intervention.

**Discussion:**

This study will evaluate whether the Vascular Navigation PAD system can reduce the number of DSAs in endovascular treatment. A reduction in DSAs may lead to lower contrast agent use and reduced radiation exposure for both patients and staff.

**Trial registration:**

The clinical trial is registered at the German Clinical Trials Register (ID: DRKS00035267). Registered on 10.Jan.2025.

## Administrative information

**Table:** 

Title {1}	Comparison of endovascular procedures of the lower extremities and iliac arteries with and without navigation assistance Vascular Navigation PAD: study protocol for a prospective multicenter randomized clinical trial
Trial registration {2a and 2b}	The clinical trial is registered at the German Clinical Trials Register (ID: DRKS00035267) 10.Jan.2025
Protocol version {3}	29.07.2025 version 1
Funding {4}	The study is funded by Brainlab AG, Munich, Germany
Author details {5a}	Masius Stephan; Gefäßchirurgie und endovaskuläre Chirurgie, Medizinische Fakultät, Universität Augsburg, Stenglinstr. 2, 86156 Augsburg, Deutschland Stephan.masius@uk-augsburg.de (corresponding author) Zerwes, Sebastian; Gefäßchirurgie und endovaskuläre Chirurgie, Medizinische Fakultät, Universität Augsburg, Stenglinstr. 2, 86156 Augsburg, Deutschland Sebastian.Zerwes@uk-augsburg.de Gombert Alexander; Universitätsklinikum Aachen, RWTH Aachen, Pauwlsstraße 30, 52074 Aachen agombert@ukaachen.de Uhl Christian; Universitätsklinikum Aachen, RWTH Aachen, Pauwlsstraße 30, 52074 Aachen cuhl@ukaachen.de Hyhlik-Dürr Alexander Gefäßchirurgie und endovaskuläre Chirurgie, Medizinische Fakultät, Universität Augsburg, Stenglinstr. 2, 86156 Augsburg, Deutschland Alexander.Hyhlik-Duerr@uk-augsburg.de
Name and contact information for the trial sponsor {5b}	Brainlab AG, Munich, Germany Mark Fingerle mark.fingerle@brainlab.com +49 89 991568 1723
Role of sponsor {5c}	The sponsor is funding the study as a PCMF study. The sponsor has no influence on data collection, statistical analysis, or writing reports. An independent monitor is hired to supervise the trial

## Introduction

### Background and rational {6a}

Peripheral artery disease (PAD) is becoming increasingly common [1]. It is characterized by stenoses or occlusions of the arteries, predominantly in the lower extremities, leading to impaired distal blood flow. Clinical manifestations range from intermittent claudication (IC) to critical limb ischemia (CLI) and arterial ulceration [2]. In patients with symptomatic PAD, revascularization may be considered, and endovascular procedures have become a central treatment option [3].

Endovascular procedures are often performed using a mobile C-arm for fluoroscopic and angiographic imaging [4]. Contemporary mobile C-arms can generate angiographic roadmaps after a digital subtraction angiography (DSA) provided that neither the patient nor the C-arm moves. These roadmaps provide a detailed representation of the patient’s vascular system, assisting in navigating through the arteries to reach the affected areas. However, previously acquired DSAs cannot generally be reused after repositioning. As a result, repeated angiography is often required, which increases radiation exposure and contrast agent administration. DSAs are known to be the primary radiation source during these procedures and contribute to the likelihood of stochastic radiation effects [5]. Because contrast agents are administered intra-arterially and may be nephrotoxic, minimizing their use is clinically relevant, particularly in a patient population with frequent comorbidities such as diabetes and reduced renal function [6].

The Vascular Navigation PAD system (Brainlab, Munich, Germany) provides image guidance during endovascular PAD procedures of the lower limbs, including the iliac arteries. It receives image data from the angiographic system, processes selected images, and constructs a vessel tree from multiple angiographic images. This enables continuous localization of endovascular devices relative to vascular anatomy by overlaying the vessel tree on live fluoroscopy. If a matching image pair is available, a repeat DSA may not be necessary after repositioning the C-arm. A CE certificate was issued in June 2024; the FDA approval is pending.

The primary aim of this study is to determine whether the investigational device reduces contrast agent volume during endovascular procedures of the lower extremities. Secondary aims are to measure radiation dose to patients and staff and to assess the device’s ability to support navigation of endovascular devices to predefined targets in PAD procedures.

This is the first clinical study of the new Vascular Navigation PAD. Retrospective studies with the EndoNaut 2D fusion imaging system (Therenva, Rennes, France), a comparable device, showed a reduction in contrast agent use [7, 8]. A randomized prospective study is therefore needed to evaluate these findings in a controlled setting.

### Objectives {7}

The study compares the investigational device with current standard care in endovascular treatment of PAD. It will evaluate whether use of the Vascular Navigation PAD system reduces the volume of contrast agent required during lower-extremity endovascular procedures. Because this is a post-market clinical follow-up study, both clinical effectiveness and safety are part of the evaluation.

#### Primary objective

The primary objective is to determine the ability of the Vascular Navigation PAD to reduce the volume of administered contrast agent compared to procedures performed without the device.

#### Secondary objectives

The secondary objectives of this study are to evaluate the following:

- Radiation dose received by patients during surgeries with and without the device
- Radiation dose received by the physicians during surgeries with and without the device
- Patient air kerma received during surgeries with and without the device
- Qualitative assessment of the device ability to enable navigation of endovascular devices to defined targets in PAD procedures

### Trial design {8}

This study is a multicenter, two-arm, prospective, confirmatory randomized superiority trial comparing a study group (navigation assistance) with a control group (procedure without navigation assistance). Patients with a clinical diagnosis of PAD who are eligible for endovascular treatment will be randomized to standard treatment or treatment with the Vascular Navigation PAD system in a 1:1 ratio.

## Methods: participants, interventions, and outcomes

### Study setting {9}

The study will be conducted in the tertiary University hospital Augsburg, Germany and the tertiary University hospital RWTH Aachen, Germany. A total of 160 patients will be recruited with equal allocation across sites and study groups.

The study population comprises adult patients with PAD and stenotic lesions in the lower extremities who are candidates for endovascular treatment.

### Eligibility criteria {10}

Below are the inclusion and exclusion criteria of this trial:

#### Inclusion criteria

- Subject has been diagnosed with peripheral arterial disease of the lower limbs or iliac arteries with Rutherford category 2–6
- Subject is eligible for endovascular surgery
- Subject provides written informed consent
- Subject is ≥18 years

#### Exclusion criteria

Subjects will be excluded from participating in this clinical investigation if one or more of the following exclusion criteria are met:

- Subject requires bypass surgery or other conventional surgery revascularization for a target lesion
- Subject has a known contrast agent allergy
- Subject is pregnant or nursing
- Subject is unable to consent

#### Performing surgeons

The interventions will be performed by a specialist in vascular surgery at the two participating hospitals.

### Who will take informed consent? {26a}

The investigator will inform potential patients both orally and in writing about the study procedures, as well as their responsibilities and rights while participating. The investigator will address all questions posed by the potential subjects. Each potential patient will sign and date two copies of the informed consent form at least 24 h before surgery. The forms will also be signed and dated by the investigator as a witness to the patient’s signature. The patient will receive one copy of the patient information, while the investigator will retain the other signed informed consent form.

### Additional consent provisions for collection and use of participant data and biological specimens {26b}

Additionally, participants of this trial can give informed consent for collecting preoperative CT-scans, but it is not mandatory for participating. The collected CT-scans are used for later comparison with intraoperative imaging. If the participants do not consent to collecting the CT-scans, the preoperative data will be used and stored in accordance with the local regulations of the treating hospital.

No biological specimens will be collected or stored for this trial.

## Interventions

### Explanation for the choice of comparators {6b}

The control group will undergo endovascular revascularization using X-ray fluoroscopy with a mobile C-arm, including roadmapping when available. This reflects standard care in German hospitals for PAD interventions, as hybrid operating rooms are not widely available. Mobile flat-panel C-arm systems will be used in both groups.

### Intervention description {11a}

No specific pre-planning procedures involving patient data are required. Patients will receive treatment according to standard clinical procedures using a mobile flat-panel C-arm. The treating physician will determine the procedural steps, including stenting if indicated. The protocol therefore focuses on the study-specific elements rather than providing a fully detailed description of routine clinical care.

Any steps that deviate from the standard of care are highlighted in bold. The subsequent section delineates, in broad terms, the intraoperative workflow with the trial device during a PAD procedure:

- The mobile flat-panel system will be started and connected via HDMI with the Brainlab hardware running Vascular Navigation PAD.
- The patient will be transported to the OR. Patient is under local, regional, or general anesthesia.
- A vascular sheath will be placed at the surgeon’s discretion.
- The surgeon will create one or more roadmaps according to the device functionality principle, by capturing DSAs and fluoroscopic images within the Vascular Navigation PAD. This capturing process is within the intended use of the investigational device, but additional to the standard of care at the study site.

Note: Image acquisition is done using a flat-panel angiographic imaging device. Acquisition of those image types is standard protocol during endovascular procedures and therefore does not add additional radiation exposure to the clinical procedure.

- Optional: The surgeon may mark areas of suspected lesions within the Vascular Navigation PAD.
- The captured images will be stitched together to generate a vessel tree. Image recognition will suggest anatomical overlap, which the surgeon may manually adjust and must confirm.
- The created (continuous) roadmap(s) will be used to support the navigation of endovascular tools to one or more lesions. The principle of single-image roadmaps is standard of care.
- The surgeon will then treat those lesions at their discretion and continue with the procedure as intended.
- The vascular access will be closed as usual (end of clinical procedure).

Post-operative:

- All image and video data in relation to the procedure (including preoperative imaging data) will be exported and uploaded to a secure platform.
- A CRF will be completed for each enrolled patient, and no additional diagnostic tests will be performed as part of the study.

### Criteria for discontinuing or modifying allocated interventions {11b}

Cross-over to Vascular Navigation PAD from state-of-the-art or vice versa is not according to protocol. A change from device group to the standard of care may at the discretion of the operating surgeon, and this information must be captured in the case report form. Reasons for the change would be the surgeon believing that the roadmap created by the Vascular Navigation PAD does not correspond to the actual vascular system.

### Strategies to improve adherence to interventions {11c}

Reasons for non-compliance could include Vascular Navigation PAD failure, surgeon error, failure to communicate correct randomization allocation to the surgeon and OR personnel, and cross-over. The patient could impact compliance if they express a wish to withdraw between randomization and surgical procedure or in the event of death.

To improve adherence, treating surgeons and OR personnel receive annual training in the navigation system PAD in accordance with the German Medical Devices Act. The study documentation, including the randomization information, is brought to the operating room with the patient and reviewed by the surgeon before the procedure begins.

### Relevant concomitant care permitted or prohibited during the trial {11d}

There is no restriction on concomitant care during the trial. Standard periprocedural anticoagulation (e.g., heparin) and antiplatelet therapy are permitted and will be documented.

### Provisions for post-trial care {30}

The trial ends with the end of the surgical procedure. There is no risk for the participants outside the standard operational risk covered by the hospital insurance.

### Outcomes {12}

#### Primary outcome

Primary efficacy parameter of the study is the final value administered contrast agent volume of a manufacturer iodine (concentration of 300 µg/mL) at the end of the procedure. The value is measured by the local research team.

The mean volume will be compared between the intervention and the control group.

#### Secondary outcome

The secondary endpoints are:

- Radiation dose received by patients, quantified in the dose-area product (DAP) (µGy·cm^2^) and cumulative air kerma (Gy)
- Radiation dose received by the physicians, quantified in microsievert (µSv) measured with a personal dosimeter worn at the chest above the lead apron vest
- Qualitative assessment of the device ability to enable navigation of endovascular devices to defined targets in PAD procedures using a 5-point Likert scale

All data shall be recorded by the OR team at the time of the procedure immediately after the sheath is removed.

In addition, the influence on the procedural time in minutes, number of DSAs, fluoroscopy time in minutes, and consumables per lesion shall be noted.

### Participation timeline {13}

Participants in this study only have two visits, screening and intervention. There are no planned follow-up visits. The details are in the study diagram:

**Table:** 

Timepoint	*t* < 0	Enrollment		Post-intervention/discharge
		0	1	2 (end of intervention)
Enrollment				
Eligibility screen	X			
Informed consent	X			
Randomization		X		
Intervention (operation with or without Vascular Navigation PAD)			X	
Assessments				
Baseline characteristics (age, weight, height)	X			
Renal function	X			
Pre-existing condition (diabetes, coronary heart disease)	X			
Contrast agent used				X
Fluoroscopy time				X
Air kerma				X
Dose-area product				X
Procedural time				X
Number of angiography series				X

### Sample size {14}

It is the aim of this study to determine the investigational device’s ability to reduce the nephrotoxic contrast agent volume required during endovascular procedures of the lower extremities. Existing clinical literature suggests a potential reduction of 30% in the volume of contrast agent when comparing procedures with navigation support to those without it [7, 9]. Additionally, information on usually needed contrast agent during procedures without navigation support is provided by a systematic review [9]. Reported values are as follows:

- Navigation-supported group = 34.7 ± 13.8 mL
- Non-navigated group (control):
  - ◦ Group 1 = 51.3 ± 26.7 mL [7])
  - ◦ Group 2 = 88.31 ± 55.35 mL (systematic review, [10])
  - ◦ Group 3 = 81.14 ± 58.68 mL (systematic review, [10])

Based on this clinical literature, the expected mean contrast volume was assumed to be 88.16 mL in the control group and 34.7 mL in the navigation-assisted group, corresponding to an anticipated absolute reduction of 53.46 mL. For sample size planning, a superiority margin of 30% of the control-group mean was prespecified, corresponding to 26.45 mL, which was used as the clinical threshold for superiority and not as the expected treatment effect. Assuming a common standard deviation of 55.27 mL, a two-sided significance level of 0.05, and a power of 90%, the required sample size was calculated for a two-group superiority design with a continuous endpoint.

After allowing for a dropout rate of 10%, 80 patients per group were planned.

### Recruitment {15}

Recruitment is carried out from patients of the participating hospitals who present electively or via the emergency room and are planned to have an endovascular procedure and meet all inclusion criteria. Currently each participating hospital performs approximately 200 endovascular procedures annually in the OR. With an estimated participation rate of 25% of the eligible patients, the calculated number of 160 patients should be reached in 10–12 months.

## Assignment of interventions: allocation

### Sequence generation {16a}

Once baseline assessments and patient enrollment are completed, participants will be randomized in a 1:1 ratio using a simple envelope randomization. No dedicated sequence generation is used in this study. The envelopes were prepared by the study office staff and are kept in a container by them following thorough mixing. The envelopes are outwardly identical. The surgeons participating in the study are not involved in the randomization process. Participants will draw their envelope out of a pool of the remaining allocations after enrollment.

### Concealment mechanism and implementation {16b} {16c}

Allocation concealment for all participants is ensured by using sequentially numbered opaque envelopes. All envelopes are prepared at the beginning of the study by a study nurse not involved in the inclusion and randomization of the patients.

Patients will be enrolled to the study by their treating physician and randomization will be performed post-consent. Participants will be randomized to Vascular Navigation PAD group or standard imaging techniques in a ratio of 1:1 post-consent and confirmation of eligibility.

## Assignment of interventions: blinding

### Blinding {17a}

Due to the nature of the intervention, it is not possible to blind the surgeon and outcome assessor. After randomization, the study team is aware of the allocation.

Emergency unblinding is not required in this study.

The data statisticians will be blinded to treatment allocation. The datasets from the two groups are handed over to the statisticians in a blinded manner for primary statistical analysis.

### Procedure for unblinding if needed {17b}

N/a; there will be no blinding in this trial.

## Data collection and management

### Data collection methods {18a}

Sites will be provided with a paper-based case report form (CRF) containing the relevant data required to be collected. Outcomes are noted by the operation team or the surgeon.

### Plans to promote participant retention and complete follow-up {18b}

No plans to promote participant retention and complete follow-up are established, as participation ends after the hospital index stay.

### Data management {19}

All study data will be handled confidentially by the investigational team and recorded using a case report form (CRF). The patient data reported in the CRFs shall be derived from source documents, be consistent with these, and any discrepancies shall be explained in writing.

### Confidentiality {27}

Any personal data will only be seen by the treating physicians and necessary hospital staff and will remain at the hospital. All data will be pseudonymized and a subject ID log will be securely stored at the hospital in a database locally in the secure hospital network before transferring to Brainlab. Only the hospital’s trained study staff has access to this information. Under no circumstances shall Brainlab have access to the subject ID log. All subjects shall consent to the processing (including the anonymization of their data by Brainlab) and transfer of their pseudonymized data to Brainlab AG and its subsidiaries.

The CRFs will be stored until at least 15 years in Brainlab’s content management system, Windchill, with restricted access until after the last Vascular Navigation PAD has been on the market. Data in Windchill will be handled according to Brainlab’s technical and organizational measures.

### Plans for collection, laboratory evaluation, and storage of biological specimens for genetic or molecular analysis in this trial/future use {33}

N/a; there will be no collection of biological specimens.

## Statistical methods

### Statistical methods for primary and secondary outcomes {20a}

The primary and secondary endpoints will be summarized as mean ± standard deviation or median, as appropriate. Because contrast agent volume and radiation dose are continuous variables and each participant contributes one observation, group comparisons will be performed using an unpaired two-sample *t*-test if the data are approximately normally distributed. The primary analysis will be conducted according to the intention-to-treat principle.

#### Survey data

The Likert scale values from survey data will be averaged over all surgeons and presented as median and interquartile range.

### Interim analyses {21b}

There will be no planned formal interim analyses of the primary and secondary outcomes, the treatment is given at baseline, and it is not possible to subsequently stop treatment for a given participant.

### Methods for additional analyses (e.g., subgroup analyses) {20b}

A subgroup analysis will be executed on all endpoints based on indication/diagnosis, lesion location, and amount and type of endovascular devices used and placed (e.g., stents).

### Methods in analysis to handle protocol non-adherence and any statistical methods to handle missing data {20c}

Reasons for non-compliance would include device failure, surgeon error, or failure to communicate correct randomization allocation to the surgeon. The patient could only impact compliance if they express a wish to withdraw between randomization and surgical procedure. Missingness will be reported and reasons for missingness explored. All acquired data until withdrawal will be used.

### Plans to give access to the full protocol, participant-level data, and statistical code {31c}

N/a; the full protocol will remain confidential.

## Oversight and monitoring

### Composition of the coordinating center and trial steering committee {5d}

The principal investigator of the trial is located at the University hospital Augsburg and coordinates the trial. There are two coinvestigators, one for each study site to coordinate the recruitment of the participants and data collection. The coinvestigators will report once a month to the principal investigator.

### Composition of the data monitoring committee, its role and reporting structure {21a}

An independent monitor is hired to partially fulfill the tasks of a data monitoring committee such as evaluation of the study documentation and completeness of the CRFs. The monitor will verify study activities and documentation compliance to study protocol and study specific procedures. CRFs will be reviewed for completeness and legibility, that corrections are completed according to GCP requirements and that they are signed appropriately.

### Adverse event reporting and harms {22}

Safety reporting in clinical investigations on medical devices shall be performed in line with the requirements of the Regulation (EU) 2017/745—Medical Device Regulation (MDR) Article 80(2). As the investigated medical device is CE marked and will be used within its intended purpose, following Article 74.1, the SAE reporting for these PMCF clinical investigations is governed by Articles 80(5) and 80(6).

Where any adverse event occurs, the relationship with the study product will be assessed to determine the relationship and be judged as causal relationship, probable, possible, or not related.

All SAEs, SARs, and SUSARs will be reported immediately (and certainly no later than 3 calendar days) by the investigator to Brainlab.

### Frequency and plans for auditing trial conduct {23}

CRF completeness and adherence to the study protocol will be checked for the first 5 included study participants and thereafter on 20% of all included study participants. All visits are intended to be on-site and according to the following plan:

- Before enrollment of the first patient
- After 1–5 patients
- After 40 patients
- After enrollment of all 80 patients
- After follow-up

Given that this study has a low-risk profile, with patients facing the same risks as they would without participating, the data monitoring is conducted by both the hospital and the sponsor.

### Plans for communicating important protocol amendments to relevant parties (e.g., trial participants, ethical committees) {25}

Brainlab will be responsible for preparing and submitting protocol amendments to the ethics committee and circulating updated document versions to recruiting study sites.

Site and principal investigators will be responsible for communicating relevant information to study participants.

### Dissemination plans {31a}

The final trial results will be published in a peer-reviewed journal.

## Discussion

This multicenter randomized trial is designed to assess whether the Vascular Navigation PAD system (Brainlab, Germany) can reduce contrast agent use during endovascular procedures of the lower extremities. By facilitating navigation and potentially reducing the need for repeated digital subtraction angiographies, the device may also decrease radiation exposure for both patients and staff. These potential benefits are clinically relevant in a PAD population that frequently presents with renal impairment and other comorbidities. As the first prospective study evaluating this technology, the trial is intended to provide high-quality evidence on its clinical utility in routine practice.

A limitation of the study is the use of the envelope randomization method. While employed for its logistical simplicity, this method carries the risk of predictability bias, as only a limited number of envelopes remain available toward the end of the study. Based on the patients already enrolled, it may be possible to infer the nature of the remaining allocations.

## Trial status

The recruitment of the first patient started on 24.02.2025. The study recruitment is planned to finish until October 2026. Current protocol is version 2 from 20.06.2025.

## Data Availability

The final trial dataset will be at the hospital site of the chief principal. Also the scanned CRFs will be stored until at least 15 years in Brainlab’s content management system, Windchill, with restricted access until after the last Vascular Navigation PAD has been on the market. Data in Windchill will be handled according to Brainlab’s technical and organizational measures.

## Abbreviations

CLI: Critical limb ischemia
CRF: Case report form
DSA: Digital subtraction angiography
EVAR: Endovascular aneurysm repair
IC: Intermittent claudication
PAD: Peripheral artery disease
CE: Conformité Européenne
FDA: Food and Drug Administration

## References

[1] Horváth L, Németh N, Fehér G, Kívés Z, Endrei D, Boncz I. Epidemiology of peripheral artery disease: narrative review. Life 2022;12(7):1041. 10.3390/life12071041 PMC9320565 35888129

[2] Soyoye DO, Abiodun OO, Ikem RT, Kolawole BA, Akintomide AO. Diabetes and peripheral artery disease: a review. World J Diabetes. 2021;12(6):827–38. 10.4239/wjd.v12.i6.827. 34168731 10.4239/wjd.v12.i6.827 PMC8192257

[3] Beckman JA, Schneider PA, Michael S. Advances in revascularization for peripheral artery disease: revascularization in PAD. Circulation Res 2021;128(12):1885–1912. 10.1161/CIRCRESAHA.121.318261 10.1161/CIRCRESAHA.121.318261 34110904

[4] Zaid Al-Kaylani AH, Schuurmann RCL, Maathuis WD, Slart RHJA, De Vries JPM, Bokkers RPH. Clinical applications of quantitative perfusion imaging with a C-arm flat-panel detector—a systematic review. Diagnostics (Basel). 2023;13(1):128. 10.3390/diagnostics13010128. 10.3390/diagnostics13010128 PMC9818280 36611421

[5] Gouëffic Y. Comparison of radiation exposure during endovascular treatment of peripheral arterial disease with flat-panel detectors on mobile C-arm versus fixed systems. Ann Vasc Surg. 2018;47:104–13. 10.1016/j.avsg.2017.08.036. 28893700 10.1016/j.avsg.2017.08.036

[6] Latus J, Schwenger V, Schlieper G, Reinecke H, Hoyer J, Persson PB, et al. Kontrastmittelinduzierte akute Nierenschädigung –Konsensuspapier derArbeitsgemeinschaft „Herz –Niere“ der Deutschen Gesellschaft für Kardiologie –Herz- und Kreislaufforschung e. V. und der Deutschen Gesellschaftfür Nephrologie e. Kardiologe. 2020;14:494–504. 10.1007/s12181-020-00411-2. 10.1007/s00108-020-00938-2 33349899

[7] Caradu C, Stenson K, Houmaïda H, Le Ny J, Lalys F, Ducasse E, et al. EndoNaut two-dimensional fusion imaging with a mobile C-arm for endovascular treatment of occlusive peripheral arterial disease. J Vasc Surg. 2021. 10.1016/j.jvs.2021.08.069. 34509588 10.1016/j.jvs.2021.08.069

[8] Malafosse C, Massiot N, Guimo F, Abdallah IB, Duprey A. Impact of the Endonaut® angio-navigation system on radiation exposure in endovascular aortic repair performed with mobile C-arms. Ann Vascular Surg. 2024;109:143-8. ISSN 0890–5096. 10.1016/j.avsg.2024.04.013. 10.1016/j.avsg.2024.04.013 38986839

[9] Lee S-R, Zhuo H, Zhang Y, Dahl N, Dardik A, Ochoa Chaar CI. Risk factors and safe contrast volume thresholds for postcontrast acute kidney injury after peripheral vascular interventions. J Vasc Surg. 2020;72(2):603–10. 10.1016/j.jvs.2019.09.059. 31843298 10.1016/j.jvs.2019.09.059

